# Lung ultrasound is non-inferior to bronchoscopy for confirmation of double-lumen endotracheal tube positioning: a randomized controlled noninferiority study

**DOI:** 10.1186/s12871-022-01707-4

**Published:** 2022-05-30

**Authors:** Sawita Kanavitoon, Kasana Raksamani, Michael P. Troy, Aphichat Suphathamwit, Punnarerk Thongcharoen, Sirilak Suksompong, Scott S. Oh

**Affiliations:** 1 Department of Anesthesiology, Faculty of Medicine Siriraj Hospital, Mahidol University, 2 Wanglang Road, Bangkoknoi, Bangkok, 10700 Thailand; 2 Division of Pulmonary, Critical Care and Sleep Medicine, Department of Medicine, David Geffen School of Medicine at UCLA, Los Angeles, CA USA; 3 Division of Cardiothoracic Surgery, Department of Surgery, Faculty of Medicine Siriraj Hospital, Mahidol University, Bangkok, Thailand

**Keywords:** Lung ultrasonography, Double lumen tube position, Lung isolation, One-lung ventilation, Fiberoptic bronchoscopy, Double lumen endotracheal tube

## Abstract

**Background:**

Appropriate placement of left-sided double-lumen endotracheal tubes (LDLTs) is paramount for optimal visualization of the operative field during thoracic surgeries that require single lung ventilation. Appropriate placement of LDLTs is therefore confirmed with fiberoptic bronchoscopy (FOB) rather than clinical assessment alone. Recent studies have demonstrated lung ultrasound (US) is superior to clinical assessment alone for confirming placement of LDLT, but no large trials have compared US to the gold standard of FOB. This noninferiority trial was devised to compare lung US with FOB for LDLT positioning and achievement of lung collapse for operative exposure.

**Methods:**

This randomized, controlled, double-blind, noninferiority trial was conducted at the Faculty of Medicine Siriraj Hospital, Mahidol University, Bangkok, Thailand from October 2017 to July 2019. The study enrolled 200 ASA classification 1–3 patients that were scheduled for elective thoracic surgery requiring placement of LDLT. Study patients were randomized into either the FOB group or the lung US group after initial blind placement of LDLT. Five patients were excluded due to protocol deviation. In the FOB group (*n* = 98), fiberoptic bronchoscopy was used to confirm lung collapse due to proper positioning of the LDLT, and to adjust the tube if necessary. In the US group (*n* = 97), lung ultrasonography of four pre-specified zones (upper and lower posterior and mid-axillary) was used to assess lung collapse and guide adjustment of the tube if necessary. The primary outcome was presence of adequate lung collapse as determined by visual grading by the attending surgeon on scale from 1 to 4. Secondary outcomes included the time needed to adjust and confirm lung collapse, the time from finishing LDLT positioning to the grading of lung collapse, and intraoperative parameters such has hypotension or hypertension, hypoxia, and hypercarbia. The patient, attending anesthesiologist, and attending thoracic surgeon were all blinded to the intervention arm.

**Results:**

The primary outcome of lung collapse by visual grading was similar between the intervention and the control groups, with 89 patients (91.8%) in the US group compared to 83 patients (84.1%) in the FOB group (*p* = 0.18) experiencing adequate collapse. This met criteria for noninferiority per protocol analysis. The median time needed to confirm and adjust LDLT position in the US group was 3 min (IQR 2–5), which was significantly shorter than the median time needed to perform the task in the FOB group (6 min, IQR 4–10) (*p* = 0.002).

**Conclusions:**

In selected patients undergoing thoracic surgery requiring LDLT, lung ultrasonography was noninferior to fiberoptic bronchoscopy in achieving adequate lung collapse and reaches the desired outcome in less time.

**Trial Registration:**

This study was registered at clinicaltrials.gov, NCT03314519, Principal investigator: Kasana Raksamani, Date of registration: 19/10/2017.

## Background

Single-lung isolation and ventilation (SLV) are of paramount importance during thoracic surgery to manage secretions, optimize exposure, and provide operative access to the area of surgical interest. Although several methods have been employed to achieve SLV, the preferred technique is placement of a left sided double lumen endotracheal tube (LDLT) [1, 2]. LDLT is considered the gold standard for SLV due to its simplicity and high rate of success in achieving lung isolation and surgical exposure [3, 4]. These high success rates rely on optimal LDLT positioning, which minimizes intraoperative complications such as hypoxia and cross-contamination of lung contents [5, 6]. The LDLT is preferred for both sides of the operations due to the simplicity of insertion. The use of FOB for checking the opening of the right upper lobe is not necessary, opposite to the right-side double-lumen tube. Double lumen tube (DLT) positioning is divided into two sequential steps: insertion of the DLT and confirmation of the DLT position [5]. Traditional methods of LDLT placement rely on blind advancement of the LDLT into the bronchus after laryngoscopic tracheal intubation. This ‘blind’ insertion technique—followed by clinical confirmation via auscultation and assessment of chest wall excursion—has excellent time efficiency and often results in acceptable placement of the LDLT [7–9].

Nevertheless, several studies suggest placement of LDLT using clinical methods alone lacks reliability, with significant variations in sensitivity and specificity and a strong effect of operator skill [7, 8, 10–12]. In contrast, fiberoptic bronchoscopy (FOB) produces more consistent results and can be used for initial placement of LDLT, assessment of appropriate depth, adjustment to optimal position within the margin of safety, and intraoperative secretion management [7, 10, 13, 14]. Therefore, FOB is considered the gold standard for confirming appropriate positioning of the LDLT and has widely supplanted the blind insertion technique in modern thoracic anesthesiology [13, 15]. Despite its advantages, a fiberoptic bronchoscope is an expensive and delicate piece of equipment that requires careful maintenance and the proper training to verify the anatomy of tracheobronchial tree [16, 17]. Moreover, from the collective experience of the authors, bronchoscopy is difficult to perform when there is bleeding in the airway.

In recent years, lung ultrasound (US) has gained attention for its ability to rapidly detect lung collapse and atelectasis with high sensitivity and specificity. Lung US easily detects collapse and absence of ventilation by loss of lung sliding [18]. Indeed, several recent studies have compared US to clinical methods for DLT positioning and found that US significantly increases accuracy of LDLT placement [11, 12, 19–21]. The limited training requirements, cost effectiveness, and simplicity of lung ultrasound make it a compelling alternative to clinical assessment for LDLT positioning, but little investigation has been performed to compare US to the current gold standard of FOB [22]. One small, prospective comparison study demonstrated excellent concordance between FOB and US findings for assessment of LDLT positioning, with analyses of time and cost effectiveness analysis favoring US [23]. Building upon these findings, we hypothesized that lung US would be non-inferior to FOB for confirming proper LDLT position and establishing SLV, thus optimizing lung collapse and surgical exposure. To test this hypothesis, a randomized, double-blind trial was devised comparing US with FOB for the primary outcome of lung collapse as graded by the operating thoracic surgeon.

## Methods

This prospective, randomized, controlled, double-blind, noninferiority trial was approved by the Institutional Review Board of the Faculty of Medicine Siriraj Hospital, Mahidol University, Bangkok, Thailand (Si 226/2017). This study was registered at clinicaltrials.gov (NCT03314519, Principal investigator: Kasana Raksamani, Date of registration: 19/10/2017) prior to the start of patient enrollment, and it adhered to Consolidated Standards of Reporting Trials (CONSORT) guidelines [24]. Written informed consent was obtained from all patients during the preoperative visit and the protocol of the study was performed in accordance with the Declaration of Helsinki.

Two hundred patients aged > 18 years with American Society of Anesthesiologists (ASA) classification 1–3 that were scheduled for elective thoracic surgery and who required a left-sided double lumen tube (LDLT) during anesthesia during October 2017 to July 2019 were enrolled. Patients having one or more of the following were excluded: anticipated difficult intubation, tracheostomy tube, concurrent pneumothorax, pleural effusion, emphysema, history of pleurodesis, and/or abnormal pulmonary function test (forced expiration volume in one second, total lung capacity, or forced vital capacity < 50% of the predicted values). Patients were randomly allocated into 2 groups using software-generated randomization (Randomization.com) and the group assignments were placed in a sealed, opaque envelope.

If regional anesthesia was indicated, epidural or spinal anesthesia, or paravertebral block was performed prior to induction of anesthesia according to the recommendations of the attending anesthesiologist. All 200 patients underwent the same standard procedure for LDLT placement. LDLT (Mallinckrodt; Covidien, Dublin, Ireland) size was determined by patient gender and height. After induction of anesthesia with propofol, fentanyl, and cisatracurium, the patient was ventilated with 100% oxygen and 1.5% to 2.5% of end-tidal concentration sevoflurane via face mask for 3 to 5 min. The patient’s trachea was then intubated with a LDLT by an anesthesiologist using laryngoscopy. After the tracheal cuff and bronchial cuff were inflated, position of the LDLT was assessed using traditional clinical methods and the position of the LDLT was adjusted until positioning was deemed appropriate. The time needed to complete the intubation process was recorded. The patient was then turned to the lateral decubitus position and the position of the LDLT was reconfirmed with auscultation. Once this step was completed, the protocol in each group was started.

**Fig. 1:**
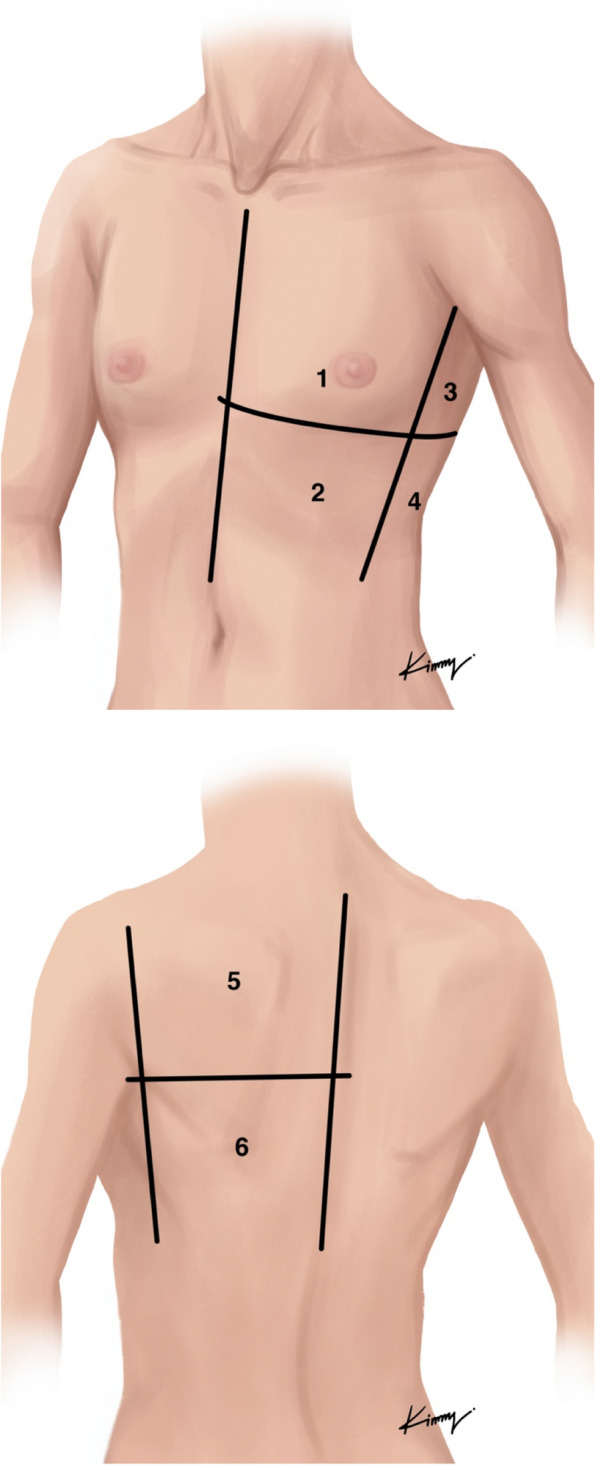
Area of scanning for lung collapse in the ultrasonography (US) group. Zones 3, 4, 5, and 6 were scanned for lung collapse in both the upper and lower lobes.

The researchers who performed FOB or US were separate personnel from the attending anesthesiologists. The attending anesthesiologists were asked to leave the operating room when the protocol started and returned after completion of the FOB or US by study faculty. Patients in the FOB group underwent fiberoptic bronchoscopy via the tracheal lumen of the LDLT to check tube position. Patients in the US group underwent lung ultrasonography using a Philips Epiq 7 ultrasound system (Philips Medical Systems, Bothell, WA, USA) with a 5–10 MHz multi-frequency linear probe to evaluate lung collapse at 4 zones (upper and lower lobe at flank and back of patient on the side of planned surgical intervention (Fig. 1), by four trained anesthesiologists [25]. The training process was theoretical instruction followed by practice scanning and direct supervision of the first 10 scans [25]. Lung collapse was defined by absence of lung sliding or pleural movement of the lung in all 4 zones. If the position of the LDLT was considered suboptimal, US or FOB was used to adjust the LDLT until the proper position was obtained, according to the randomization arm. The contralateral lung was also ultrasound in all zones to confirm lung movement from lung sliding. After final positioning by the research anesthesiologist, another attending anesthesiologist returned for the remainder of the case.

After appropriate positioning of the LDLT, the patient was painted and draped in a sterile fashion. Immediately after entering the chest wall, thoracic surgeons who were blinded to the patient’s group assignment graded the status of the lung, having been trained to give a visual scale grading of lung collapse. Grading ranged from 1 to 4, as follows: 1 = total collapse of the lung with no movement, 2 = partial collapse of the lung without movement, 3 = partial collapse of the lung with movement, and 4 = no collapse of the lung.

Three time points were recorded, as follows: 1) time from start of intubation to final patient positioning; 2) time from final patient positioning to final LDLT adjustment; and 3) time from final LDLT positioning to time of surgeon lung collapse grade. Intraoperative parameters such as hypoxia, hypercarbia, hypertension, and hypotension were recorded for each arm.

The primary outcome of this study was the dichotomized presence of adequate lung collapse by the attending thoracic surgeons according to the grading scale above, with grades 1 and 2 defined as *collapse* and grades 3 and 4 defined as *no collapse*. The secondary outcomes included the time needed to adjust and confirm lung collapse, the time from finishing LDLT positioning to the grading of lung collapse, and intraoperative parameters.

### Sample size calculation and statistical analysis

Sample size calculation was performed by n4Studies [26]based on the hypothesis that lung ultrasonography is not inferior to fiberoptic bronchoscopy (noninferiority study) [12, 27, 28]. Using a non-inferiority margin of 10%, a *p*-value less than 0.05, and power of 90%, the minimum sample size was calculated to be 82 patients. To compensate for withdrawal from the study for any cause, the size of each group was increased to 100 patients.

All statistical analyses were performed using SPSS Statistics version 21 for Windows (SPSS, Inc., Chicago, IL, USA). Continuous variables with normal distribution are presented as mean ± standard deviation, and non-normally distributed continuous variables are reported as median and interquartile range. Categorical data are shown as number and percentage. Data comparisons were performed using independent *t*-test, Mann–Whitney U test, or Pearson’s chi-squared test. A *p*-value less than 0.05 was considered statistically significant. Positive predictive value (PPV) was calculated to determine the effectiveness of lung ultrasound compared to fiberoptic bronchoscopy. Per-protocol noninferiority analysis was performed in accordance with CONSORT recommendations for noninferiority trials [24].

## Results

**Fig. 2:**
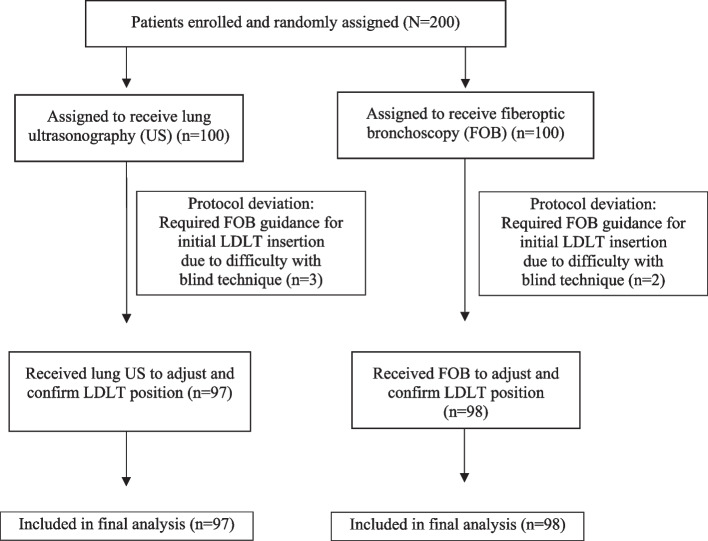
CONSORT flow diagram of the study protocol

A CONSORT flow diagram summarizing the study protocol is shown in Fig. 2. Two hundred patients were initially enrolled and randomized; however, 5 patients were withdrawn from the study due to protocol deviation, (3 in the US group and 2 in the FOB group, all of whom required FOB for initial positioning of ETT prior to onset of study protocol portion of the procedure). The remaining 97 patients in the US group and the remaining 98 patients in the FOB group completed the study and were included in the final analysis. There was no significant difference between groups in baseline characteristics including age, gender, height, weight, body mass index, primary diagnosis, or type of surgery (Table 1). There was also no significant difference between groups for comorbidities, except that there was significantly more hypertension in US patients than in FOB patients (54 [55.7%] *vs.* 38 [38.8%], respectively; *p* = 0.02). Grading of lung collapse by the attending surgeon was categorized as *collapse* (total collapse and partial collapse, no movement) or *no collapse* (partial collapse with lung movement and no lung collapse). Eighty-nine patients (91.8%) and 83 patients (84.7%) demonstrated lung collapse by visual grading in the US group and the FOB group, respectively (Table 2). The PPV in the US group was 91.8% versus 84.7% in the FOB group (*p* = 0.182, 95% confidence interval [CI]: -16.9% to 2.7%). The original grading was 51 patients (52.6%) with total collapse in the US group versus 52 patients (53.1%) in the FOB group, and 38 patients (39.2%) versus 31 patients (31.6%) with partial collapse, no lung movement in the US group and the FOB group, respectively. In the ultrasound group, 2 patients (2%) demonstrated partial collapse with movement and 6 patients (6.2%) demonstrated no collapse. In the FOB group, 13 patients (13.3%) were graded as partial collapse with movement and 2 patients (2%) demonstrated no collapse.

**Table 1:** Patient demographic and clinical characteristics

Characteristics	Group US (*n* = 97)	Group FOB (*n* = 98)	*p*-value
Baseline characteristics			
Age (yrs)	60.4 ± 15.6	60.6 ± 12.8	0.93
Male gender	38 (39.2%)	48 (49.0%)	0.19
Height (cm)	158 ± 8.8	160 ± 7.8	0.16
Weight (kg)	61.4 ± 10.9	62.1 ± 12.7	0.65
Body mass index (kg/m^2^)	24.4 ± 4.4	24.0 ± 4.3	0.58
ASA physical status			0.12
1	5 (5.2%)	11 (11.2%)	
2	59 (60.8%)	64 (65.3%)	
3	33 (34.0%)	23 (23.5%)	
Primary diagnosis			0.64
Lung nodule/mass	59 (60.8%)	65 (66.3%)	
Lung cancer	24 (24.7%)	25 (25.5%)	
Lung bleb	1 (1.0%)	0 (0.0%)	
Pneumothorax (minimal residual air)	2 (2.1%)	1 (1.0%)	
Others	11 (11.3%)	7 (7.1%)	
Comorbidity			
Diabetes mellitus	18 (18.6%)	13 (13.3%)	0.34
Hypertension	54 (55.7%)	38 (38.8%)	***0.02***
Dyslipidemia	19 (19.6%)	18 (18.4%)	0.86
Other	24 (24.7%)	14 (14.3%)	
Type of surgery			0.60
Video-assisted thoracoscopic surgery	73 (75.3%)	74 (75.5%)	
Thoracotomy	24 (24.7%)	23(23.5%)	
Median sternotomy	0 (0.0%)	1 (1.0%)	
Size of double lumen tube			0.38
32	4 (4.1%)	3 (3.1%)	
35	56 (57.7%)	48 (49.0%)	
37	37 (38.1%)	47 (48.0%)	

Data presented as number and percentage or mean ± standard deviation

A *p*-value < 0.05 indicates statistical significance

**Abbreviations**: *US* ultrasonography, *FOB* fiberoptic bronchoscopy, *ASA* American Society of Anesthesiologists

**Table 2:** Visual grading of lung collapse in 195 patients

Group	Visual grading by surgeon	
	**Collapse**	**No collapse**
Ultrasonography (*n* = 97)	89 (91.8%)	8 (8.2%)
Fiberoptic bronchoscopy (*n* = 98)	83 (84.7%)	15 (15.3%)

**Fig. 3:**
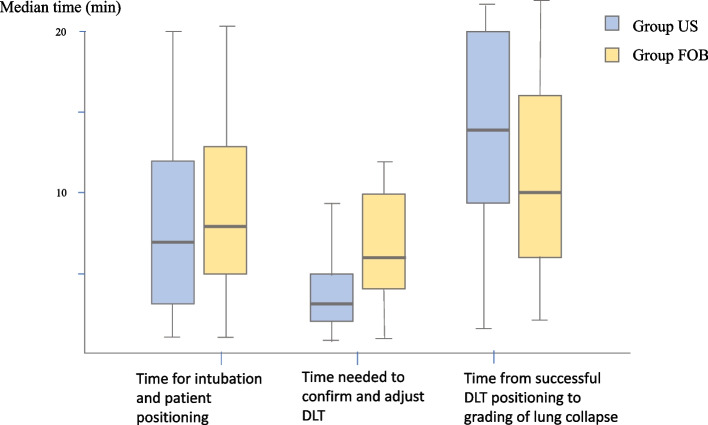
Median time for each procedural step in the ultrasonography (US) and fiberoptic bronchoscopy (FOB) groups (Abbreviation: DLT, double lumen tube)

The median times needed to perform defined procedural steps compared between groups are presented in Fig. 3. The median time needed for intubation and patient positioning was 7 min [Interquartile range (IQR): 3–12] and 8 min [IQR: 5–13] in the US and FOB groups, respectively (*p* = 0.18). The median time needed to confirm and adjust LDLT position was 3 min [IQR: 2–5] in the US group and 6 min [IQR: 4–10] in the FOB group (*p* < 0.001). Time from final LDLT positioning to visual grading of lung collapse was 14 min [IQR: 9.5–20] and 10 min [IQR: 5.9–16] in the US group and the FOB group, respectively (*p* = 0.002). There was no significant difference for these time parameters within groups when we compared between lung collapse and no lung collapse (Table 3).

**Table 3:** Time to accomplish defined procedural steps in collapse and no collapse patients compared between the US group and the FOB group

	**Group US** **(*n* = 97)**			**Group FOB** **(*n* = 98)**		
	**Collapse (*n* = 89)**	**not collapse (*n* = 8)**	***p*-value**	**Collapse (*n* = 83)**	**not collapse (*n* = 15)**	***p-*value**
Time for initial intubation and patient positioning (minutes)	7 (3–12)	9 (5–14.25)	0.47	8 (5–13)	8 (5–10)	0.56
Time needed to confirm and adjust LDLT (minutes)	3 (2–5)	5 (1.39–13)	0.41	6 (4–10)	6 (5–9)	0.69
Time from successful LDLT positioning to grading of lung collapse (minutes)	14 (9.5–20)	14.5 (5–18)	0.77	9 (5–14.3)	16 (11–32)	0.002

Data reported as median and interquartile range

A *p*-value < 0.05 indicates statistical significance

Abbreviations: *US* ultrasonography, *FOB* fiberoptic bronchoscopy, *LDLT* left sided double lumen endotracheal tube

There was no significant difference in complications between the US and FOB groups. Twenty-five patients (25.7%) versus 21 patients (21.4%) developed intraoperative hypertension (greater than 20% increase in blood pressure from baseline) in the US and FOB groups, respectively (*p* = 0.5), and the mean duration was less than 2 min. Thirty-three patients (34%) in the US group developed hypotension (greater than 20% decrease in blood pressure from baseline), while 29 patients (29.6%) developed hypotension in the FOB group (*p* = 0.5). The mean duration of hypotension was less than 3 min in both groups. Oxygen desaturation was defined in this study as oxygen saturation less than 90% at any time and desaturation was observed in 16 patients (16.5%) in the US group compared to 11 patients (11.2%) in the FOB group (*p* = 0.29), with a mean duration of 10.9 and 8.4 s, respectively.

## Discussion

This randomized controlled study revealed assessment of LDLT placement via lung US to be noninferior to FOB for achieving adequate collapse of the target lung. Time to adjust LDLT position was significantly shorter by lung US than by FOB. Incidence of intraoperative complication was not significantly different between groups, confirming the safety of lung US for solely use in confirmation of the LDLT position.

The current gold standard technique is blind insertion of the LDLT with subsequent confirmation by FOB [8, 13]. However, in emergency situations, especially when inserting a DLT in scenarios complicated by bleeding or infection outside the operating theater, blind insertion and clinical auscultation remains an effective alternative method [29, 30]. Our study demonstrated high accuracy of lung US for adjusting and confirming lung collapse from proper LDLT position, which suggests that it may be used as an alternative method to confirm LDLT position, with FOB kept available as back-up for situations where there is any uncertainty or difficulty with placement.

Bronchoscopy provides direct visualization of the bronchial anatomy and bronchial cuff position, yielding benefit in patients with abnormal anatomy and in difficult airway situations [26, 27]. FOB is also effective for guiding the insertion of the LDLT from the beginning of the intubation procedure in certain situations [4, 31]. In this study, we had 5 patients with protocol deviation that were excluded due to difficulties with initial insertion of the LDLT, and all 5 of these patients required FOB to guide the insertion. Moreover, FOB can be used to suction secretions and blood in the airway that can obstruct the inside of the LDLT, which can result in proper LDLT position, but no lung collapse [26].

Nevertheless, US provides key advantages, including wide availability, ease of use, and significantly decreased exposure to respiratory secretions, thus minimizing operator risk in situations where the patient has transmissible respiratory pathogens [22, 25]. This study demonstrated that the time needed for confirmation and adjustment of the LDLT position was significantly shorter when using lung US compared to when using FOB (median time 3 vs. 6 min, respectively), reducing potential exposure time even further. Additionally, the use of US may be highly valued in resource-limited settings where minimizing use of FOB may result in significant cost savings.

### Limitations

This study has some notable limitations. First, in contrast to FOB, lung US is not able to directly visualize LDLT position. Although this was not observed during our trial, the LDLT might not be in the perfect position even though lung US detects lung collapse, which may lead to LDLT malposition during the operation. Second, the ultrasonographer could not be completely blinded due to the clinical visualization of the chest rising after intubation before and during US. Third, although the pattern of the US evaluation was classified using the upper and lower portions of the lung, grading was unified to the whole lung as collapse, partial collapse, or no collapse. Fourth, the lung might be properly collapsed by blind technique. Assessment of subsequently collapse by lung US or FOB could be affected. These factors could result in biases relating to the assessment of lung collapse.

Finally, this study was limited to left-sided DLT and excluded patients with ASA class 4 or 5 and those with pre-existing lung pathology such as pneumothorax, effusion, or emphysema. The strengths of this study are its randomized prospective design, the robustness of its blinding process, and the use of a clinically relevant primary endpoint rather than a surrogate.

## Conclusions

The results of this study showed lung US to be non-inferior to FOB for achieving adequate lung collapse from proper double lumen endotracheal tube placement, as determined by surgical grading. Moreover, the time needed to confirm and adjust LDLT position was significantly shorter with US compared to FOB. This study suggests that using US in a stepwise manner may allow clinicians to avoid the need for FOB in every LDLT placement, instead reserving FOB for more difficult initial intubations or those where US fails to demonstrate collapse. Decreased use of bronchoscopy has benefits such as limiting exposure to potential respiratory pathogens and optimizing resource use. In addition to use in controlled operative settings, this method may be beneficial to use along with FOB for confirming double lumen tube position and lung isolation in emergent or infectious settings.

## Data Availability

The datasets generated and/or analysed during the current study are not publicly available due as this may compromise participant anonymity but are available from the corresponding author on reasonable request.
