# Correction: Lung ultrasound is non-inferior to bronchoscopy for confirmation of double-lumen endotracheal tube positioning: a randomized controlled noninferiority study

**DOI:** 10.1186/s12871-024-02409-9

**Published:** 2024-01-23

**Authors:** Sawita Kanavitoon, Kasana Raksamani, Michael P. Troy, Aphichat Suphathamwit, Punnarerk Thongcharoen, Sirilak Suksompong, Scott S. Oh

**Affiliations:** 1 Department of Anesthesiology, Faculty of Medicine Siriraj Hospital, Mahidol University, 2 Wanglang Road, Bangkoknoi, Bangkok, 10700 Thailand; 2 Division of Pulmonary, Critical Care and Sleep Medicine, Department of Medicine, David Geffen School of Medicine at UCLA, Los Angeles, CA USA; 3 Division of Cardiothoracic Surgery, Department of Surgery, Faculty of Medicine Siriraj Hospital, Mahidol University, Bangkok, Thailand

**Correction: BMC Anesthesiol 22, 168 (2022)**

10.1186/s12871-022-01707-4.

Following publication of the original article [1], the authors reported an error to the Institutional Review Board (IRB) number found in both the Methods and Ethics approval and consent to participate sections. The incorrect IRB number specified in the published paper is “088/2017” and the correct IRB number should be “Si 226/2017”.

The original article [1] has been updated.
